# MDDI-SCL: predicting multi-type drug-drug interactions via supervised contrastive learning

**DOI:** 10.1186/s13321-022-00659-8

**Published:** 2022-11-15

**Authors:** Shenggeng Lin, Weizhi Chen, Gengwang Chen, Songchi Zhou, Dong-Qing Wei, Yi Xiong

**Affiliations:** 1 State Key Laboratory of Microbial Metabolism, Shanghai-Islamabad-Belgrade Joint Innovation Center on Antibacterial Resistances, Joint International Research Laboratory of Metabolic & Developmental Sciences and School of Life Sciences and Biotechnology, Shanghai Jiao Tong University, Shanghai, 200240 China; 2 Zhongjing Research and Industrialization Institute of Chinese Medicine, Nanyang, 473006 China; 3 Peng Cheng National Laboratory, Shenzhen, 518055 China; 4 Shanghai Artificial Intelligence Laboratory, Shanghai, 200232 China

**Keywords:** Drug-drug interaction, Multi-type classification, Supervised contrastive learning, Multi-scale feature fusion, Self-attention mechanism

## Abstract

The joint use of multiple drugs may cause unintended drug-drug interactions (DDIs) and result in adverse consequence to the patients. Accurate identification of DDI types can not only provide hints to avoid these accidental events, but also elaborate the underlying mechanisms by how DDIs occur. Several computational methods have been proposed for multi-type DDI prediction, but room remains for improvement in prediction performance. In this study, we propose a supervised contrastive learning based method, MDDI-SCL, implemented by three-level loss functions, to predict multi-type DDIs. MDDI-SCL is mainly composed of three modules: drug feature encoder and mean squared error loss module, drug latent feature fusion and supervised contrastive loss module, multi-type DDI prediction and classification loss module. The drug feature encoder and mean squared error loss module uses self-attention mechanism and autoencoder to learn drug-level latent features. The drug latent feature fusion and supervised contrastive loss module uses multi-scale feature fusion to learn drug pair-level latent features. The prediction and classification loss module predicts DDI types of each drug pair. We evaluate MDDI-SCL on three different tasks of two datasets. Experimental results demonstrate that MDDI-SCL achieves better or comparable performance as the state-of-the-art methods. Furthermore, the effectiveness of supervised contrastive learning is validated by ablation experiment, and the feasibility of MDDI-SCL is supported by case studies. The source codes are available at https://github.com/ShenggengLin/MDDI-SCL.

**Supplementary Information:**

The online version contains supplementary material available at 10.1186/s13321-022-00659-8.

## Introduction

The use of multiple drugs, often termed as polypharmacy, is a therapeutic approach to treat various complex diseases [1, 2]. However, polypharmacy can lead to drug-drug interactions (DDIs), in which the pharmacological effect of a drug is altered by another drugs [3–5]. It has been estimated that DDIs are associated with 30% of all the reported adverse drug events (ADEs) which may result in the majority of incidence and mortality, and even drug withdrawal from the market, incurring huge medical expense due to the stringent demands on drug development [6]. Therefore, it is necessary to reliably identify DDIs and understand their underlying mechanisms, which will be beneficial for drug development in pharmaceutical companies and can provide important information on polypharmacy prescription for clinicians and patients. In vitro experiments and clinical trials can be conducted to identify DDIs, but systematic combinatorial screening of DDI candidates from a large pool of drugs by experimental techniques remains challenging, time- and resource-consuming.

In the last decades, there are increasing availability of scientific literature, electronic medical records, population-based reports of adverse events, drug labels, and other related sources [7]. Researchers attempted to extract DDIs from scientific literature and electronic medical records via natural language processing (NLP) techniques [8, 9], infer potential DDIs by similarity-based methods based on known DDIs [10], and predict DDIs by leveraging machine learning [11], network modelling [12, 13], and knowledge graphs [14, 15]. However, most of these computational methods (except the extraction of DDIs via NLP methods) only consider whether a DDI occurs or not given a pair of drugs.

To facilitate the understanding of the causal mechanisms of DDIs, recent studies have developed multi-type DDIs prediction methods to elaborate sufficient details beyond the chance of DDI occurrence [16]. The pioneering study by Ryu et al. constructed the gold standard DDI dataset from DrugBank [17], which covers 192,284 DDIs associated with 86 DDI types (changes in pharmacological effects and/or the risk of ADEs as a result of DDI) from 191,878 drug pairs [18]. Then, they formulated the multi-type DDI prediction as a multi-label classification task and proposed DeepDDI by using deep neural network (DNN) based on structural information of chemical compounds for a drug pair. This architecture became a baseline for several other state-of-the-art multi-type DDI prediction methods, which improved the multi-type DDI prediction by incorporating various types of biological information such as drug targets and enzymes to represent a drug pair in addition to the structural information of drugs based on autoencoder or the encoder module of transformer for learning the low-dimensional latent features and DNN algorithms for classification [19–21]. It should be noted that those methods represent the feature vector of a drug by the similarity profile, which is generated by the similarity (i.e., structural similarity) of a given drug against each one in the rest of drugs across the entire dataset. More recently, Deng et al. used few-shot learning based on the latent features from a pair of drug structures to improve the prediction performance on rare types of DDIs which have few samples [22]. Liu et al. proposed the method CSMDDI, which first generates the embedding representations of drugs and DDI types and then learns a mapping function to bridge the drugs attributes to their embeddings to predict multi-type DDIs [23]. Feng et al. proposed deepMDDI, which consists of an encoder by deep relational graph convolutional networks constraining with similarity regularization to capture the topological features of DDI network and a tensor-like decoder for multi-label prediction of DDI types [24]. Yang et al. proposed a substructure-aware graph neural network, utilizing a message-passing neural network with a novel substructure attention mechanism and a substructure-substructure interaction module for DDI prediction [25].

With the increasing availability of large biomedical knowledge graphs (KGs), some studies attempt to incorporate KG with other data (i.e., drug molecular structures) for multi-type DDI predictions via graph neural networks (GNNs) [26, 27]. However, there are data redundancy and noise in the large KGs, in which only a small subgraph is relevant to a prediction target [28, 29]. Thus, the KG-based prediction methods for DDIs are still at the infant stage.

Although these published methods have achieved some success in multi-type DDI prediction, there still exist some limitations. First, datasets of DDI types are extremely unbalanced, and these methods have poor performance in predicting rare types with fewer samples. Second, most methods perform well in predicting unknown DDI types between known drugs, but they often fail to do it for new drugs. It will be useful to develop the new methods to resolve the problems and further improve the prediction performance.

Since the labelled data is limited and expensive to obtain, contrastive learning has recently become a popular and powerful strategy to get quality representations of samples in a self-supervised way. It aims at embedding augmented versions of the same sample close to each other while trying to push away embeddings from different samples [30]. Contrastive learning is not only used for self-supervised tasks, but also for supervised tasks. Khosla et al. extend the self-supervised batch contrastive approach to the fully-supervised setting, allowing models to effectively leverage label information [31]. For supervised contrastive learning, the samples belonging to the same class are pulled together in embedding space, while simultaneously pushing apart samples from different classes [31, 32].

**Fig. 1:**
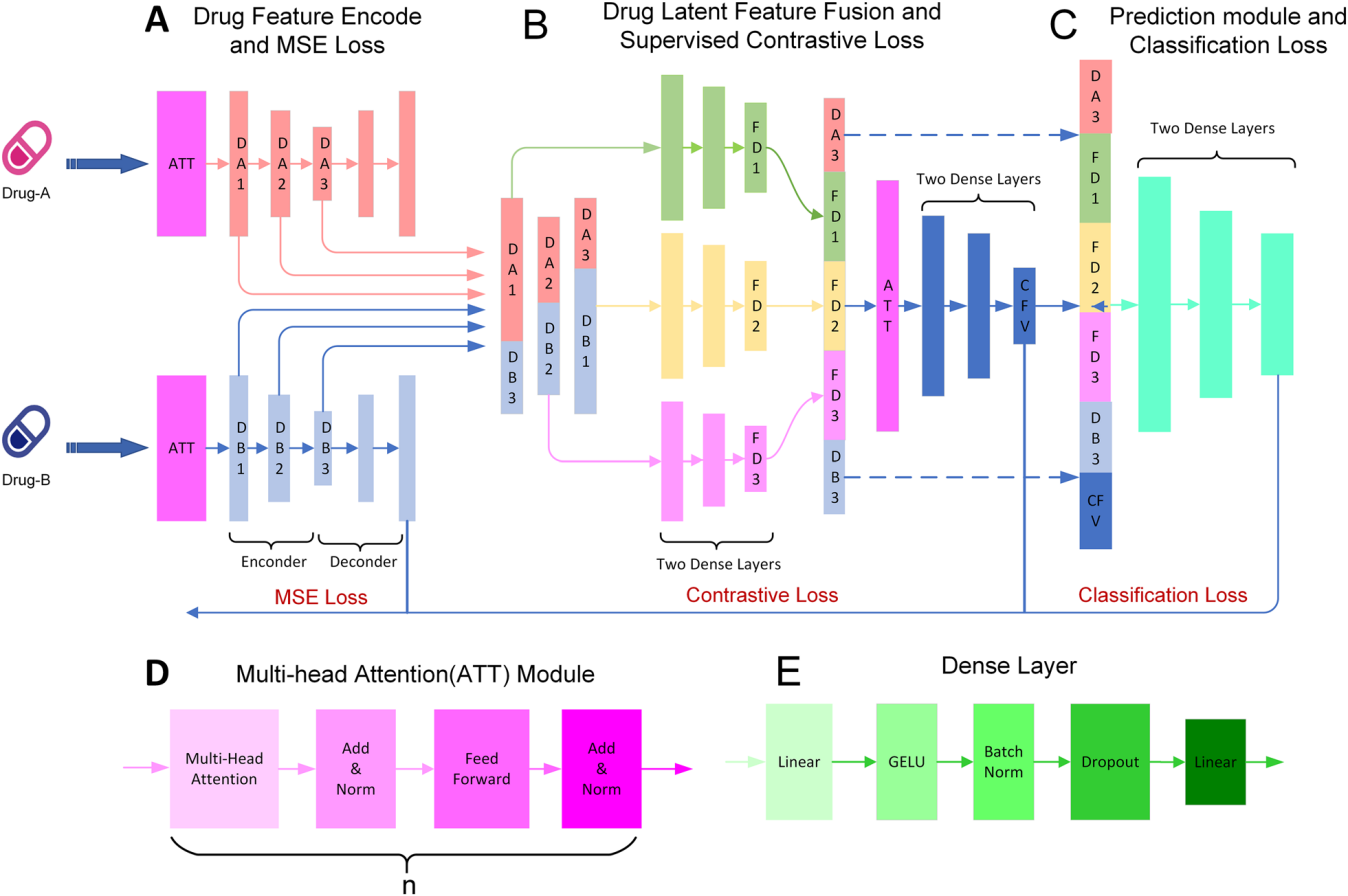
The overview of the proposed MDDI-SCL method. **A** Drug feature encode and MSE loss module. **B** Drug latent feature fusion and supervised contrastive loss module. **C** Multi-type DDIs prediction and classification loss module. **D** Multi-head Attention (ATT) module. **E** Dense layer module

Contrastive learning has been successfully applied in the field of bioinformatics [33–38]. In this study, we propose a new method named MDDI-SCL for multi-type DDI prediction, which is based on Supervised Contrastive Learning (SCL) and three-level loss functions. MDDI-SCL (Fig. 1) mainly includes three parts: drug feature encoder and mean squared error (MSE) loss module, drug latent feature fusion and supervised contrastive loss module, DDI type prediction and classification loss module. Specifically, we first input the drugs into the drug encoder to obtain the lower-dimensional latent features of each drug by MSE. Then, the latent features of two drugs are combined as input into the feature fusion module to obtain the latent features of the drug pairs. Supervised contrastive loss can make the features of the same type of DDIs more similar, and the features of DDIs from different types more different. Therefore, we can obtain features that are more powerful to classification by using contrastive loss in the feature fusion module. Finally, we input the latent features of each drug pair into the multi-type DDI prediction module to predict DDI types, and update the model parameters by the classification loss.

Experimental results demonstrate that MDDI-SCL achieves better performance than several state-of-the-art methods on all three tasks of two different datasets. Additionly, we also proved the effectiveness of supervised contrastive learning for multi-type DDI prediction. More importantly, results of the case studies validated the feasibility of our method in practice.

## Materials and methods

### Datasets

In this study, we use two datasets with the number of samples at a different scale. The first dataset (Dataset1) is the benchmark dataset that Deng et al. collected [20]. Dataset1 contains 572 drugs with 74, 528 pairwise DDIs, which are associated with 65 DDI types. Each drug in Dataset1 has four types of features: chemical substructures, targets, pathways and enzymes, which are extracted from DrugBank [39]. The second dataset (Dataset2) is the dataset from the study of Lin et al. [21]. Dataset2 contains 1, 258 drugs with 323, 539 pairwise DDIs, which are associated with 100 DDI types. Each drug in Dataset2 has three types of features: substructures, targets and enzymes.

### Drug feature representation

Each feature type of a drug corresponds to a set of descriptors, so one drug can be represented by a binary feature vector, and its value (1 or 0) indicates the presence or absence of the corresponding element.

These feature vectors have high dimensionality with values of most of dimensions being 0. Therefore, we represent the feature vector of a drug by the similarity profile, which is generated by the similarity of drug A against each one (i.e., drug B) in the rest of drugs in the dataset [18]. Jaccard similarity is calculated by the following equation,

1 $$\mathbf{J}\mathbf{a}\mathbf{c}\mathbf{c}\mathbf{a}\mathbf{r}\mathbf{d}\left(\mathbf{A},\mathbf{B}\right)=\frac{\left|\mathbf{A}\cap \mathbf{B}\right|}{\left|\mathbf{A}\cup \mathbf{B}\right|}=\frac{\left|\mathbf{A}\cap \mathbf{B}\right|}{\left|\mathbf{A}\right|+\left|\mathbf{B}\right|-\left|\mathbf{A}\cap \mathbf{B}\right|}$$

where A and B are original bit vectors of two drugs; |A ∩ B| is the number of elements in the intersection of A and B; |A ∪ B| is the number of elements in the union of A and B.

Based on the Jaccard similarity, in Dataset1, each type feature of a drug is represented as a 572-dimensional vector. Therefore, each drug with four type of features is represented by a 4*572-dimentional vector. In the similar way, each drug is represented as a 3*1258-dimensional vector in Dataset2.

### Drug feature encoder and mean squared error loss

The drug feature encoder module mainly includes multi-head self-attention layers and an autoencoder. The multi-head self-attention layers can focus on more important drug features [40, 41], and further the autoencoder performs feature dimensionality reduction [42, 43]. Consequently, lower-dimensional and better drug representations can be obtained through the drug feature encoder module. We use mean squared error loss to update the parameters of the feature encoder module.

#### Multi-head self-attention mechanism and autoencoder

The detailed description of the multi-head self-attention mechanism and autoencoder is provided in the Additional file 1 [41]. In the model, the hidden features obtained through the multi-head self-attention layers are denoted as DA1 and DB1 for a pair of drugs (i.e., drug A and drug B), as shown in Fig. 1A. The encoder of autoencoder has two linear layers. The output vectors of the first linear layer are denoted as DA2 and DB2, and the output vectors of the second linear layer are denoted as DA3 and DB3.

#### Mean squared error

Mean squared error is commonly used as regression loss function, which calculates average squared difference between the observed and predicted values. In our model, MSE is the sum of squared distances between the drug feature vector and the output vector of decoder divided by the feature dimensionality. The MSE is calculated by following formula,

2 $$\mathrm{MSE}=\frac{\sum_{\mathrm{i}=1}^{\mathrm{fea}\_\mathrm{dim}}{({\mathrm{val}}_{\mathrm{i}}-{\mathrm{val}}_{\mathrm{i}}^{\sim })}^{2}}{\mathrm{fea}\_\mathrm{dim}}$$

where *fea_dim* is the feature dimensionality of the drug, *val*_*i*_ is the value of each dimension of the drug feature vector, *val*_*i*_^~^ is the value of each dimension of the output vector of the decoder.

### Drug latent feature fusion and supervised contrastive loss

The drug latent feature fusion module mainly includes two sub-modules: multi-scale feature fusion and latent feature dimensionality reduction. The multi-scale feature fusion sub-module can simultaneously combine the low-level features and high-level features of a drug pair, and the feature dimensionality reduction sub-module can further fuse latent features and reduce the feature dimensionality. The supervised contrastive learning loss function is utilized to update the parameters of the drug latent feature fusion module.

#### Multi-scale feature fusion sub-module

A drug pair contains two drugs (i.e., drug A and drug B). Through the drug feature encoder module, three latent features of drug A are obtained: DA1, DA2, and DA3, as shown in Fig. 1A. Similarly, we can acquire three latent features of drug B: DB1, DB2, and DB3. DA1 and DB1 are low-level features, which usually contain more detailed information but also more noise [44, 45]. DA3 and DB3 are high-level features. Normally, high-level features have more semantic information and less noise but lose a lot of detailed information [45–48]. Thus, in order to better integrate the advantages of low-level features and high-level features, we concatenate DA1 and DB3, DA2 and DB2, DA3 and DB1 to represent a drug pair, respectively. Then, we input the concatenated features into the fully connected layer to obtain the fused drug pair features FD1, FD2, and FD3, as shown in Fig. 1B.

#### Latent feature dimension reduction sub-module

When the neural network becomes deep, residual connection can be used to avoid the problem of vanishing gradient [ 49 ]. In this sub-module, the output (DA3 and DB3) of encoder and the output (FD1, FD2 and FD3) of multi-scale feature fusion sub-module are concatenated as input into the latent feature dimensionality reduction sub-module, which mainly includes multi-head self-attention layers and linear layers. The number of neurons for each linear layer is half of the former layer. Multi-head self-attention has been introduced in detail in “Multi-head self-attention mechanism and autoencoder” section.  The output vector of latent feature dimensionality reduction sub-module is named CFV, as shown in Fig. 1B.

#### Supervised contrastive loss

Contrastive learning includes unsupervised contrastive learning and supervised contrastive learning. The latent features of samples obtained by unsupervised contrastive learning have the following property: the features of samples from the same source are more similar, whereas the features of samples from different sources are more different [50]. However, one significant disadvantage of unsupervised contrastive learning is that it does not consider the correlation of features between samples from different sources yet belonging to the same class. To overcome this drawback of unsupervised contrastive learning, supervised contrastive learning is proposed. The latent features of samples obtained by supervised contrastive learning have the following property: the features of samples belonging to same type are more similar, while the features of samples of different types are more different [31, 51].

Considering that the DDI type prediction task is a multi-class classification task, supervised contrastive learning is more competent for this task. Accordingly, our model employs supervised contrastive learning. The loss function of supervised comparative learning in our model can be calculated by the following formula,

3 $${{\varvec{l}}}^{{\varvec{c}}{\varvec{o}}{\varvec{n}}}=\frac{1}{{{\varvec{N}}}_{{\varvec{b}}{\varvec{a}}{\varvec{t}}{\varvec{c}}{\varvec{h}}{\varvec{s}}{\varvec{i}}{\varvec{z}}{\varvec{e}}}}\sum_{{\varvec{i}}=1}^{{{\varvec{N}}}_{{\varvec{b}}{\varvec{a}}{\varvec{t}}{\varvec{c}}{\varvec{h}}{\varvec{s}}{\varvec{i}}{\varvec{z}}{\varvec{e}}}}{{\varvec{l}}}_{{\varvec{i}}}^{{\varvec{c}}{\varvec{o}}{\varvec{n}}}$$

4 $${{\varvec{l}}}_{{\varvec{i}}}^{{\varvec{c}}{\varvec{o}}{\varvec{n}}}=\frac{-1}{{{\varvec{N}}}_{{{\varvec{y}}}_{{\varvec{i}}}}-1}\sum_{{\varvec{j}}=1,{\varvec{j}}\ne {\varvec{i}},{{\varvec{y}}}_{{\varvec{j}}}={{\varvec{y}}}_{{\varvec{i}}}}^{{{\varvec{N}}}_{{\varvec{b}}{\varvec{a}}{\varvec{t}}{\varvec{c}}{\varvec{h}}{\varvec{s}}{\varvec{i}}{\varvec{z}}{\varvec{e}}}}{\varvec{l}}{\varvec{o}}{\varvec{g}}\frac{{\varvec{e}}{\varvec{x}}{\varvec{p}}({\varvec{s}}{\varvec{i}}{\varvec{m}}({{\varvec{C}}{\varvec{F}}{\varvec{V}}}_{{\varvec{i}} },{{\varvec{C}}{\varvec{F}}{\varvec{V}}}_{{\varvec{j}}})/{\varvec{\tau}})}{\sum_{{\varvec{k}}=1,{\varvec{k}}\ne {\varvec{i}}}^{{{\varvec{N}}}_{{\varvec{b}}{\varvec{a}}{\varvec{t}}{\varvec{c}}{\varvec{h}}{\varvec{s}}{\varvec{i}}{\varvec{z}}{\varvec{e}}}}{\varvec{e}}{\varvec{x}}{\varvec{p}}({\varvec{s}}{\varvec{i}}{\varvec{m}}({{\varvec{C}}{\varvec{F}}{\varvec{V}}}_{{\varvec{i}}} , {{\varvec{C}}{\varvec{F}}{\varvec{V}}}_{{\varvec{k}}})/{\varvec{\tau}})}$$

where *N*_*batchsize*_ is the number of samples in each batch, *y*_*i*_ is the class label of sample *i*, and *y*_*j*_ is the class label of sample *j*. *N*_*yi*_ is the number of samples of class *y*_*i*_ in the same batch. *sim* is a function that measures the similarity of two vectors, such as cosine similarity. *CFV*_*i*_*, CFV*_*j*_*, CFV*_*k*_ are the latent feature vector, which are the output vector of latent feature dimensionality reduction sub-module of sample *i*, *j*, and *k*, respectively. *τ ∈ R*^+^ is a scalar temperature parameter. According to the above formulas, in order to make the *l*_*i*_^*con*^ loss smaller, the value of *sim(CFV*_*i,*_* CFV*_*j*_*)* will be larger. So the hidden vectors *CFV*_*i*_ and *CFV*_*j*_ must be more similar. *CFV*_*i*_ and *CFV*_*j*_ are the latent vectors of the same type samples, so the latent features of the same type samples are more similar.

### Multi-type DDI prediction and classification loss

The module employs two fully connected layers to predict DDI types, and the number of neurons in the second fully connected layer is the number of DDI types. DDI type prediction is a multi-class classification task, and the sample size of each class is not balanced. Since focal loss can partially solve the problem of sample imbalance [21], we use focal loss [52] and cross-entropy loss as our classification loss functions. In detail, we choose the cross-entropy loss as our classification loss function in the first one third of training steps, and apply focal loss as our classification loss function in the last two thirds of steps. Therefore, the total loss function of the model is as follows:

5 $$\mathrm{Loss}={\mathrm{l}}_{\mathrm{MSE}}(\mathrm{x},{\mathrm{x}}^{\sim })+{\mathrm{l}}_{\mathrm{con}}(\mathrm{CFV},\mathrm{y})+{\mathrm{l}}_{\mathrm{cla}}(\mathrm{y},{\mathrm{y}}^{\sim })$$,

where *x* is the feature vector of the drug pair, *x* ~ is the output vector of the decoder, *CFV* is the output vector of latent feature dimensionality reduction sub-module, *y* is the class label of sample, and *y* ~ is the predicted value of sample. *l*_*MSE*_ is MSE loss function, *l*_*con*_ is supervised contrastive learning loss function and *l*_*cla*_ is classification loss function. *l*_*cla*_ is composed of the cross-entropy loss in the first one third of training steps and focal loss in the last two thirds of steps.

In order to prevent over-fitting, the label smoothing strategy is implemented [53]. For multi-classification problems, the class label vector is often converted into one-hot vector. However, the one-hot vector may weaken the generalization ability of the model and result in over-fitting. Label smoothing uses the smoothing parameter to add noise to the one-hot encoding, making the model less confident about its predictions. Therefore, it can partially solve the problem of over-fitting.

We utilize Gaussian error linear unit activation function and Radam optimizer [54]. The dropout layer and batch normalization layer are placed between the fully connected layers [55].

## Results and discussion

### Experimental settings of prediction tasks

This study evaluated the multi-type DDI prediction tasks based on three experimental settings: (i) prediction of unobserved interaction types between known drugs (Task1); (ii) prediction of interaction types between known drugs and new drugs (Task2) and (iii) prediction of interaction types between new drugs (Task3). New drugs in the corresponding task are missing in the training set, but exist in the test set.

For Task1, we apply five-fold cross-validation (5-CV) to DDI types and split all DDI types into five subsets. We train models based on DDI types in the training set, and then make predictions for DDI types in the test set. For Task2 and Task3, we apply 5-CV to drugs instead of DDI types. We randomly split drugs into five subsets, and used four of them as training drugs, leaving the remaining one as test drugs. For Task2, prediction models are constructed on the DDI types between two training drugs, and then make predictions for DDI types between training drugs and test drugs. For Task3, prediction models are built on the DDI types between two drugs in the training set to predict for DDI types between two drugs in the test set.

For model evaluation, accuracy (ACC), area under the precision-recall-curve (AUPR), area under the ROC curve (AUC), F1 score, precision and recall are adopted as evaluation metrics. On highly imbalanced data sets, AUPR and F1 score metrics are more objective for model evaluation. Consequently, in the following discussion, we will focus on these two metrics.

### Hyper-parameters setting

**Fig. 2:**
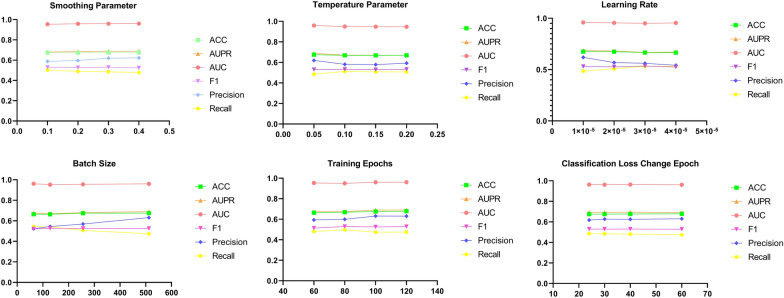
The prediction performance of six hyper-parameters settings on Task2 of Dataset1

The chosen of hyper-parameters influences the performance of model. First, we discussed the settings of  six hyper-parameters on affecting the prediction performance on Task2 of Dataset1: smoothing parameter in the label smoothing strategy, temperature parameter in the contrastive learning, learning rate, batch size, training epochs and the epoch to change the cross-entropy loss to focal loss. Task1 is a relatively simple task, while Task3 is a relatively difficult task. Thus, to ensure the versatility of the hyper-parameters, we chose Task2 to tune the hyper-parameters. For Task1 and Task3, we used the optimal parameters tuned on Task2. The performance metrics under different settings are shown in Fig. 2.

According to Fig. 2, the performance of the model does not change drastically as the hyper-parameters change. Almost all metric scores vary within the range of 0.01. This also illustrates the stability of our model. In the end, we chose 0.3 for smoothing parameter, 0.05 for temperature parameter, 2e-5 for learning rate, 512 for batch size, 120 for training epochs and the 40^th^ epoch to change the cross-entropy loss to focal loss.

### The prediction effect of multi-scale feature fusion

**Fig. 3:**
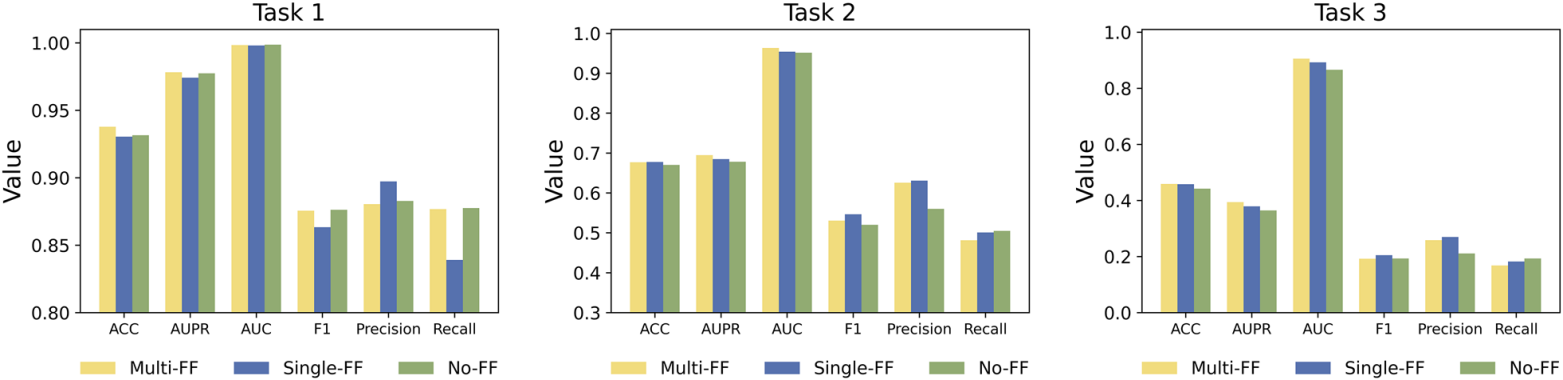
The prediction performance of different feature fusion methods on three tasks of Dataset1

In the drug latent feature fusion module, we tried three types of feature fusion methods. The first method is the single-scale feature fusion, which concatenates DA1 and DB1, DA2 and DB2, DA3 and DB3 as three assemblies. The second method is multi-scale feature fusion. Correspondingly, we concatenate DA1 and DB3, DA2 and DB2, DA3 and DB1 as three assemblies. The third method is to use only DA3 and DB3 without feature fusion. We compared these three feature fusion methods on three tasks of Dataset1, as shown in Fig. 3.

On three tasks, the AUPR and AUC of the multi-scale feature fusion method achieved the highest scores. In general, the performance of the multi-scale feature fusion method is slightly better than the other two methods. Therefore, multi-scale feature fusion is incorporated into the final model.

### The prediction effect of supervised contrastive learning

In order to verify the effectiveness of supervised contrastive learning, we compared the performance of the model with and without supervised contrastive learning on three tasks of Dataset1, as shown in Table 1. The model with supervised contrastive learning achieved better performance in ACC, AUPR, and AUC on all three tasks. The AUPR of the model with supervised contrastive learning on Task2 is 0.6947 while the AUPR of the model without supervised contrastive learning on Task2 is 0.6765. The AUC of the model with supervised contrastive learning on Task3 is 0.0313 higher than that without supervised contrastive learning. In general, model with supervised contrastive learning achieves better prediction performance.

**Table 1:** The prediction effect of supervised contrastive learning on three tasks of Dataset1

	ACC	AUPR	AUC	F1	Precision	Recall
Task1						
With SCL	0.9378	0.9782	0.9983	0.8755	0.8804	0.8767
Without SCL	0.9308	0.9746	0.9982	0.8712	0.8762	0.8752
Task2						
With SCL	0.6767	0.6947	0.9634	0.5304	0.6254	0.4814
Without SCL	0.6667	0.6765	0.9513	0.5314	0.5685	0.5177
Task3						
With SCL	0.4589	0.3938	0.9053	0.1919	0.2585	0.1678
Without SCL	0.4553	0.3772	0.8740	0.2273	0.2571	0.2177

### The prediction effect of focal loss

**Fig. 4:**
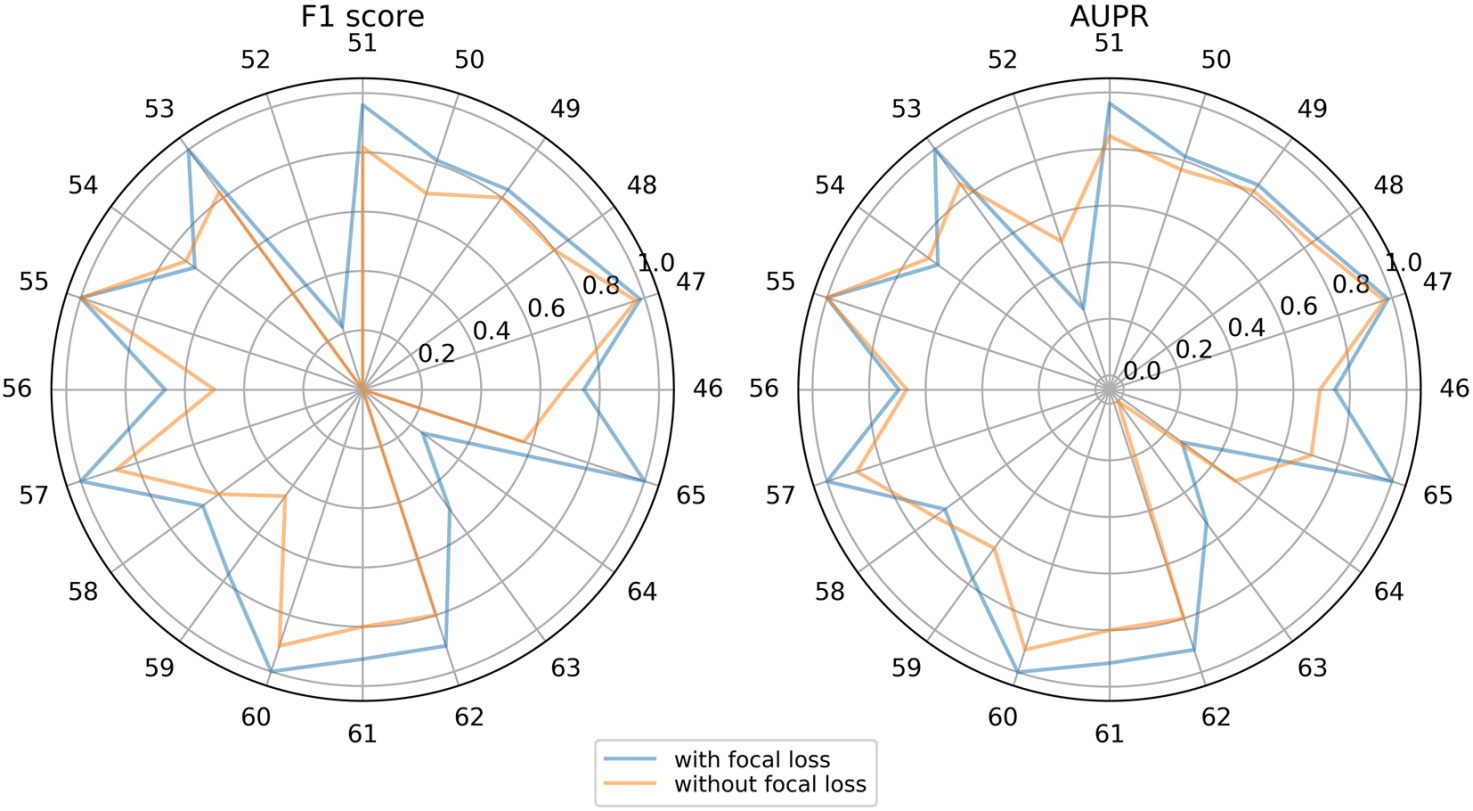
The F1 scores and AUPR scores of 20 categories with a small sample size with/without focal loss on Task1 of Dataset1

Focal loss can solve problems of imbalance in sample size of each category and difficulty of imbalanced classification. Focal loss improves the classification ability of the model by forcing the model to focus on categories with a small sample size. In order to examine whether focal loss improves the prediction for categories with small sample size, we selected 20 categories with the smallest sample size (from DDI type46 to DDI type65) on Task1 of Dataset1 for comparison, as shown in Fig. 4.

On categories with a small sample size, focal loss can boost the classification performance of the model. Among the 20 categories with a small sample size, the F1 score of the model with focal loss is higher than that of the model without focal loss on 19 categories. On DDI type 52, 63, and 64, the F1 score of the model without focal loss is 0, while the F1 score of the model with focal loss is 0.2222, 0.5, and 0.25, respectively. Among the 20 categories with a small sample size, the AUPR of the model with focal loss is higher than the AUPR of the model without focal loss on 16 categories. On DDI type 63, the AUPR of the model without focal loss is 0.0001, while the AUPR of the model with focal loss is 0.5334.

### The prediction effect of label smoothing strategy

We verified the effectiveness of the label smoothing strategy on three tasks of Dataset1. The experimental results are shown in Table 2.

**Table 2:** The prediction effect of label smoothing (LS) strategy on three tasks of Dataset1

	ACC	AUPR	AUC	F1	Precision	Recall
Task1						
With LS	0.9378	0.9782	0.9983	0.8755	0.8804	0.8767
Without LS	0.9377	0.9776	0.9981	0.8840	0.8718	0.9023
Task2						
With LS	0.6767	0.6947	0.9634	0.5304	0.6254	0.4814
Without LS	0.6659	0.6705	0.9470	0.5120	0.5275	0.5243
Task3						
With LS	0.4589	0.3938	0.9053	0.1919	0.2585	0.1678
Without LS	0.4449	0.3636	0.8723	0.1971	0.2022	0.2063

On all three tasks, the AUPR of the model using label smoothing is higher than that of the model which does not utilize label smoothing. The AUPR of the model using label smoothing on Task2 is 0.0242 higher than that without label smoothing. The AUPR of the model using label smoothing on Task3 is 0.0302 higher than that without label smoothing.

### Comparison with state-of-the-art DDI type prediction and baseline methods

We compared MDDI-SCL with other four state-of-the-art DDI type prediction methods: DeepDDI [18], Lee et al.’s methods [19], DDIMDL [20] and MDF-SA-DDI [21], and also several baseline classification methods: fully connected DNN, random forest (RF), k-nearest neighbor (KNN) and logistic regression (LR). The performance comparison of all prediction models on Dataset1 and Dataset2 is shown in Table 3 and Table 4, respectively.

**Table 3:** Performance comparison with the state-of-the-art methods on three tasks of Dataset1

	ACC	AUPR	AUC	F1	Precision	Recall
Task1						
MDDI-SCL	0.9378	0.9782	0.9983	0.8755	0.8804	0.8767
MDF-SA-DDI	0.9301	0.9737	0.9989	0.8878	0.9085	0.8760
DDIMDL	0.8852	0.9208	0.9976	0.7585	0.8471	0.7182
Lee et al.'s methods	0.9094	0.9562	0.9961	0.8391	0.8509	0.8339
DeepDDI	0.8371	0.8899	0.9961	0.6848	0.7275	0.6611
DNN	0.8797	0.9134	0.9963	0.7223	0.8047	0.7027
RF	0.7775	0.8349	0.9956	0.5936	0.7893	0.5161
KNN	0.7214	0.7716	0.9813	0.4831	0.7174	0.4081
LR	0.7920	0.8400	0.9960	0.5948	0.7437	0.5236
Task2						
MDDI-SCL	0.6767	0.6947	0.9634	0.5304	0.6254	0.4814
MDF-SA-DDI	0.6633	0.6776	0.9497	0.5584	0.6547	0.5078
DDIMDL	0.6415	0.6558	0.9799	0.4460	0.5607	0.4319
Lee et al.'s methods	0.6405	0.6244	0.9247	0.5039	0.5388	0.4891
DeepDDI	0.5774	0.5594	0.9575	0.3416	0.3630	0.3890
DNN	0.6239	0.6361	0.9796	0.2997	0.4237	0.2840
Task3						
MDDI-SCL	0.4589	0.3938	0.9053	0.1919	0.2585	0.1678
MDF-SA-DDI	0.4338	0.3873	0.8630	0.2329	0.2715	0.2226
DDIMDL	0.4075	0.3635	0.9512	0.1590	0.2408	0.1452
Lee et al.'s methods	0.4097	0.3184	0.8302	0.2022	0.2216	0.2027
DeepDDI	0.3602	0.2781	0.9059	0.1373	0.1586	0.1450
DNN	0.4087	0.3776	0.9550	0.1152	0.1836	0.1093

**Table 4:** Performance comparison with the state-of-the-art methods on three tasks of Dataset2

	ACC	AUPR	AUC	F1	Precision	Recall
Task1						
MDDI-SCL	0.9516	0.9862	0.9995	0.9321	0.9162	0.9500
MDF-SA-DDI	0.9291	0.9773	0.9996	0.9117	0.9381	0.8910
DDIMDL	0.9229	0.9637	0.9993	0.9105	0.9212	0.9039
Lee et al.'s methods	0.9370	0.9791	0.9991	0.9181	0.9226	0.9153
DeepDDI	0.7211	0.7724	0.9914	0.6854	0.6654	0.7183
DNN	0.7908	0.8539	0.9949	0.7649	0.7560	0.8046
RF	0.6956	0.7567	0.9892	0.5760	0.6694	0.5426
KNN	0.5797	0.5964	0.8998	0.3805	0.4758	0.3347
LR	0.5229	0.5288	0.9805	0.2373	0.3128	0.2185
Task2						
MDDI-SCL	0.6595	0.6794	0.9757	0.5578	0.5605	0.5712
MDF-SA-DDI	0.6664	0.6820	0.9862	0.5919	0.6526	0.5518
DDIMDL	0.6720	0.7086	0.9885	0.5817	0.6680	0.5295
Lee et al.'s methods	0.6917	0.7119	0.9687	0.5934	0.6144	0.5848
DeepDDI	0.5883	0.5851	0.9746	0.4709	0.5250	0.4361
DNN	0.6687	0.6838	0.9818	0.6164	0.7279	0.5479
Task3						
MDDI-SCL	0.4696	0.4261	0.9315	0.2838	0.3160	0.2773
MDF-SA-DDI	0.4794	0.4450	0.9686	0.2937	0.3667	0.2659
DDIMDL	0.4699	0.4386	0.9685	0.3032	0.3773	0.2729
Lee et al.'s methods	0.4867	0.4349	0.9093	0.3082	0.3355	0.3066
DeepDDI	0.3611	0.2820	0.9264	0.1868	0.2301	0.1711
DNN	0.4570	0.4129	0.9565	0.2997	0.4345	0.2508

We evaluated the performance of all prediction methods for Task1. Experimental results show that MDDI-SCL and MDF-SA-DDI perform much better than other methods on Task1 on Dataset1. MDDI-SCL achieves the best AUPR 0.9782. On Dataset2, the performance of MDDI-SCL is better than other methods. The AUPR, F1 score and ACC of MDDI-SCL is 0.9862, 0.9321 and 0.9516, respectively. These evaluation scores of MDDI-SCL are higher than that of other methods.

We also compared the state-of-the-art methods on Task2 and Task3 of the two datasets. Experimental results show that our method MDDI-SCL achieves better or comparable performance than the state-of-the-art methods on some evaluation metrics. On Dataset1, the AUPR of MDDI-SCL is 0.6947 and 0.3938 on Task2 and Task3, respectively. The AUC of MDDI-SCL is 0.6767 and 0.4589 on Task2 and Task3, respectively. These evaluation scores of MDDI-SCL are higher than that of other methods. The F1 score of MDDI-SCL is slightly worse than the state-of-the-art methods. It should be emphasized that we used the same hyper-parameters on different tasks and different datasets. We did not optimize the hyper-parameters of the model across all the datasets and tasks. The hyper-parameters of the deep learning model may affect the performance of the model, so the experimental results presented here may not be the optimal performance of our model.

In general, our model achieves better or similar performance on Task1 of both datasets compared to the state-of-the-art methods. Our model also achieves better or similar performance as the state-of-the-art methods on Task2 and Task3 of Dataset1. Our model performs slightly worse than the state-of-the-art models on Task2 and Task3 of Dataset2. This may be explained by the fact that the hyper-parameters of our model are obtained on Dataset1. Inappropriate hyper-parameters may affect the performance of the model.

### Case studies

The evaluation metrics have proved the effectiveness of our model. We conducted case studies to further validate the effectiveness of MDDI-SCL in practice.

We used all the DDI type samples on Dataset1 originally obtained from DrugBank [17] to train the prediction model, and then predicted the drug-drug pairs that do not exist on Dataset1. We focused on the five most frequent DDI types and checked up the top 20 predictions related to each type. We used the interactions checker tool provided by https://go.drugbank.com/drugs to verify these predictions.

Among 100 samples, 43 DDI type samples were confirmed, which are shown in Additional file 1: Table S1. For example, the interaction between Donepezil and Armodafinil is predicted to cause the DDI type #0, which means that metabolism of Donepezil can be decreased when combined with Armodafinil.

Under the same experimental setup, 43 of the 100 DDI samples predicted by MDDI-SCL were confirmed, whereas 35 of the 100 DDI samples predicted by MDF-SA-DDI were confirmed. This shows that MDDI-SCL is more effective than MDF-SA-DDI in practice. In Additional file 1: Table S2, we list the other 57 drug pairs among the 100 DDI samples. These drug pairs may not be reported in the literature, but these DDIs are likely to occur when taken together, which may be helpful for pharmaceutical research.

## Conclusions

We proposed a multi-type DDI prediction model based on supervised contrastive learning and three-level loss functions, and proved the effectiveness and robustness of our model. In addition, we also proved the prediction effect of supervised contrastive learning, focal loss and label smoothing strategy. Experimental results demonstrate that our proposed model achieves better or comparable performance than that of the state-of-the-art models. The case studies were also performed to identify the new DDIs which are not included in the current datasets. Moreover, the effectiveness of our model is supported by case studies in practice.

## Supplementary Information

**Additional file 1: Table S1.** Forty-three DDI samples have been confirmed among the 100 DDI samples predicted by MDDI-SCL. **Table S2.** Fifty-seven DDI samples that may not be reported in the literature among the 100 DDI samples predicted by MDDI-SCL.

## Data Availability

The source codes are available at https://github.com/ShenggengLin/MDDI-SCL. The datasets are available at https://github.com/ShenggengLin/MDF-SA-DDI.

## Abbreviations

DDIs: Drug-drug interactions
ADEs: Adverse drug events
NLP: Natural language processing
DNN: Deep neural network
KGs: Knowledge graphs
GNNs: Graph neural networks
SCL: Supervised contrastive learning
MSE: Mean squared error
ATT: Attention
ACC: Accuracy
AUPR: Area under the precision-recall-curve
AUC: Area under the ROC curve
LS: Label smoothing
RF: Random forest
KNN: K-nearest neighbor
LR: Logistic regression
