# Supplementary material for: MDDI-SCL: predicting multi-type drug-drug interactions via supervised contrastive learning
Source: J Cheminform. 2022 Nov 15;14:81. doi: 10.1186/s13321-022-00659-8 (PMC9667597; doi:10.1186/s13321-022-00659-8)
Supplement: Supplementary file 1 — Additional file 1: Table S1. Forty-three DDI samples have been confirmed among the 100 DDI samples predicted by MDDI-SCL. Table S2. Fifty-seven DDI samples that may not be reported in the literature among the 100 DDI samples predicted by MDDI-SCL. [file 13321_2022_659_MOESM1_ESM.pdf]

Supplementary Material

MDDI-SCL: predicting multi-type drug-drug interactions via supervised contrastive learning

Shenggeng Lin [a] , Weizhi Chen [a] , Gengwang Chen [a], Songchi Zhou [a] , Dong-Qing Wei [a,b,c,*] and Yi Xiong [a,d,**]

a. State Key Laboratory of Microbial Metabolism, Shanghai-Islamabad-Belgrade Joint Innovation Center on Antibacterial Resistances, Joint International Research Laboratory of Metabolic & Developmental Sciences and School of Life Sciences and Biotechnology, Shanghai Jiao Tong University, Shanghai, 200240, China.

b. Zhongjing Research and Industrialization Institute of Chinese Medicine, Nanyang, 473006, China.

c. Peng Cheng National Laboratory, Shenzhen, 518055, China.

d. Shanghai Artificial Intelligence Laboratory, Shanghai, 200232, China.

* Corresponding author.

** Corresponding author. State Key Laboratory of Microbial Metabolism, Shanghai-Islamabad-Belgrade Joint Innovation Center on Antibacterial Resistances, Joint International Research Laboratory of Metabolic & Developmental Sciences and School of Life Sciences and Biotechnology, Shanghai Jiao Tong University, Shanghai, 200240, China.

Email address: xiongyi@sjtu.edu.cn (Y. Xiong), dqwei@sjtu.edu.cn (D.-Q. Wei).

1. Multi-head self-attention mechanism

Since some features are redundant or less important, multi-head self-attention mechanism can help the model select significant features, and give these important features with higher weights for multi-type DDI prediction. Therefore, multi-head self-attention mechanism can help the model to identify which features are crucial for prediction. The multi-head attention is calculated by following formulas,

$$X_{att} = Concat(Head_1, Head_2, \ldots, Head_m)W^O \quad (1)$$

$$Head_i = softmax(\frac{Q_i \times K_i^T}{\sqrt{d_k}})V_i \quad (2)$$

$$Q_i = X \times W_i^Q \quad (3)$$

$$K_i = X \times W_i^K \tag{4}$$

$$V_i = X \times W_i^V \tag{5}$$

, where X is the drug feature vector. $W_i^Q \in R^{d_{in} \times d_Q}$, $W_i^K \in R^{d_{in} \times d_K}$, $W_i^V \in R^{d_{in} \times d_V}$ are the parameter matrices. Q_i , K_i and V_i are the Q(Query), K(Key) and V(Value) matrices derived from the linear transformation of X, respectively.

2. **Autoencoder**

Autoencoder is an unsupervised neural network model, which includes two parts: encoder and decoder. The function of the encoder is to encode high-dimensional features into low-dimensional latent features to let the neural network to learn the most informative features. The objective of a decoder is to map back the latent features into a reconstruction of the original input. The best scenario is that the output of the decoder can perfectly or approximately approach the original input. The autoencoder with single linear layer is calculated by following formulas,

$$Feature_{hidden} = g_{en}(X_{input}) = \sigma(W_{en}X_{input} + b_{en}) \tag{6}$$

$$X_{input}^{\sim} = g_{de}(Feature_{hidden}) = \sigma(W_{de}Feature_{hidden} + b_{de}) \tag{7}$$

, where X_{input} is the input feature vector of the autoencoder, and W_{en} and b_{en} are the parameters of the encoder. σ is the activation function. $Feature_{hidden}$ is the latent feature vector. W_{de} and b_{de} are the parameters of the decoder. $X_{input}^{\sim}$ is the output of the decoder.

Table S1. Forty-three DDI samples have been confirmed among the 100 DDI samples predicted by MDDI-SCL.

Drug	Drug	DDI
Armodafinil	Donepezil	The metabolism of Donepezil

		can be decreased when combined with Armodafinil.
Escitalopram	Dexlansoprazole	The metabolism of Escitalopram can be decreased when combined with Dexlansoprazole.
Memantine	Isoniazid	Isoniazid may decrease the excretion rate of Memantine which could result in a higher serum level.
Cilostazol	Clonidine	The metabolism of Clonidine can be decreased when combined with Cilostazol.
Cyclosporine	Memantine	Cyclosporine may decrease the excretion rate of Memantine which could result in a higher serum level.
Gemfibrozil	Lobeglitazone	The metabolism of Lobeglitazone can be decreased when combined with Gemfibrozil.
Miconazole	Ifosfamide	The metabolism of Ifosfamide can be decreased when combined with Miconazole.
Dosulepin	Lobeglitazone	Dosulepin may decrease the hypoglycemic activities of Lobeglitazone.
Isoniazid	Celecoxib	The metabolism of Celecoxib can be decreased when combined with Isoniazid.
Mycophenolic acid	Atomoxetine	Atomoxetine may decrease the excretion rate of Mycophenolic acid which could result in a higher serum level.
Ketoconazole	Lobeglitazone	The metabolism of Lobeglitazone can be decreased when combined with Ketoconazole.
Nicardipine	Lobeglitazone	The metabolism of Lobeglitazone can be decreased when combined with Nicardipine.

Dabigatran etexilate	Brivaracetam	Brivaracetam may decrease the excretion rate of Dabigatran etexilate which could result in a higher serum level.
Miconazole	Doconexent	The metabolism of Doconexent can be decreased when combined with Miconazole.
Isoniazid	Dosulepin	The risk or severity of serotonin syndrome can be increased when Isoniazid is combined with Dosulepin.
Carbinoxamine	Cariprazine	The risk or severity of adverse effects can be increased when Carbinoxamine is combined with Cariprazine.
Imipramine	Methylergometrine	The risk or severity of hypertension can be increased when Methylergometrine is combined with Imipramine.
Aranidipine	Amylnitrite	Aranidipine may increase the vasodilatory activities of Amyl Nitrite.
Bortezomib	Hydroxyurea	The risk or severity of adverse effects can be increased when Bortezomib is combined with Hydroxyurea.
Arsenic trioxide	Naldemedine	Arsenic trioxide may decrease the excretion rate of Naldemedine which could result in a higher serum level.
Etacrynicacid	Diazoxide	The risk or severity of adverse effects can be increased when Etacrynic acid is combined with Diazoxide.
Lidocaine	Artenimol	The metabolism of Lidocaine can be decreased when combined with Artenimol.
Debrisoquine	Artenimol	The metabolism of

		Debrisoquine can be decreased when combined with Artenimol.
Daunorubicin	Alfuzosin	The metabolism of Alfuzosin can be decreased when combined with Daunorubicin.
Epinephrine	Mifepristone	The therapeutic efficacy of Mifepristone can be decreased when used in combination with Epinephrine.
Mycophenolic acid	Bosutinib	The risk or severity of adverse effects can be increased when Mycophenolic acid is combined with Bosutinib.
Doconexent	Isavuconazole	The metabolism of Doconexent can be decreased when combined with Isavuconazole.
Hydroxychloroquine	Cilostazol	The risk or severity of QTc prolongation can be increased when Cilostazol is combined with Hydroxychloroquine.
Lorpiprazole	Benzatropine	Benzatropine may decrease the excretion rate of Lorpiprazole which could result in a higher serum level.
Granisetron	Brexpiprazole	The risk or severity of adverse effects can be increased when Granisetron is combined with Brexpiprazole.
Fexofenadine	Eltrombopag	The excretion of Fexofenadine can be decreased when combined with Eltrombopag.
Imipramine	Artenimol	The metabolism of Imipramine can be decreased when combined with Artenimol.
Fluvoxamine	Acetazolamide	The risk or severity of

		adverse effects can be increased when Acetazolamide is combined with Fluvoxamine.
Agomelatine	Cyproterone acetate	The metabolism of Agomelatine can be increased when combined with Cyproterone acetate.
Mycophenolic acid	Apalutamide	Mycophenolic acid may decrease the excretion rate of Apalutamide which could result in a higher serum level.
Lobeglitazone	Lumacaftor	The metabolism of Lobeglitazone can be increased when combined with Lumacaftor.
Danazol	Bosentan	The metabolism of Bosentan can be decreased when combined with Danazol.
Mycophenolate mofetil	Cyproterone acetate	The serum concentration of Cyproterone acetate can be decreased when it is combined with Mycophenolate mofetil.
Diethylstilbestrol	Acetohexamide	The therapeutic efficacy of Acetohexamide can be decreased when used in combination with Diethylstilbestrol.
Chlorpromazine	Miglitol	The therapeutic efficacy of Miglitol can be decreased when used in combination with Chlorpromazine.
Flurazepam	Estradiol valerate	Flurazepam may decrease the excretion rate of Estradiol valerate which could result in a higher serum level.
Levosalbutamol	Lubiprostone	Lubiprostone may decrease the excretion rate of Levosalbutamol which could result in a higher serum level.
Diethylstilbestrol	Glipizide	The therapeutic efficacy of Glipizide can be decreased when used in combination

with Diethylstilbestrol.

Table S2. Fifty-seven DDI samples that may not be reported in the literature among the 100 DDI samples predicted by MDDI-SCL.

Drug	Drug
Brigatinib	Mexiletine
Carisoprodol	Epoprostenol
Dabigatran etexilate	Crisaborole
Eslicarbazepine acetate	Alogliptin
Nabilone	Armodafinil
Minaprine	Calcium carbimide
Capsaicin	Calcium carbimide
Escitalopram	Calcium carbimide
Maprotiline	Amifostine
Lamotrigine	Calcium carbimide
Chloroprocaine	Docetaxel
Donepezil	Calcium carbimide
Fluphenazine	Desmopressin
Glycerin	Calcium carbimide
Hydralazine	Calcium carbimide
Ambenonium	Calcium carbimide
Clofarabine	Mesoridazine
Guanfacine	Calcium carbimide
Lidocaine	Calcium carbimide
Hydralazine	Artenimol
Diethylstilbestrol	Lorpiprazole
Arsenic trioxide	Netupitant
Eltrombopag	Brexpiprazole
Albendazole	Ibrutinib
Miltefosine	Cilostazol
Cevimeline	Bosutinib
Daunorubicin	Artenimol
Droxidopa	Lumacaftor
Lomustine	Cyproterone acetate
Methotrimeprazine	Apalutamide
Brigatinib	Lornoxicam
Glucosamine	Mitotane
Glucosamine	Enzalutamide
Griseofulvin	Bosentan
Mycophenolic acid	Dabrafenib
Masoprocol	Apalutamide
Amrubicin	Apalutamide

Atropine	Apalutamide
Lomustine	Dabrafenib
Meclizine	Cyproterone acetate
Nabumetone	Mitotane
Ethopropazine	Apalutamide
Lomustine	Cysteamine
Chlorpropamide	Hydrocortamate
Acetylcholine	Glimepiride
Glyburide	Mefloquine
Furosemide	Cysteamine
Lubiprostone	Hydrocortamate
Lomustine	Naltrexone
Daunorubicin	Cysteamine
Dorzolamide	Miglitol
Estramustine	Mitiglinide
Mometasone	Cysteamine
Diethylstilbestrol	Mitiglinide
Mefloquine	Acitretin
Levonorgestrel	Cysteamine
Acetylsalicylic acid	Codeine
